# Myotonic Dystrophy-1 Complicated by Factor-V (Leiden) Mutation

**DOI:** 10.1155/2015/271639

**Published:** 2015-03-30

**Authors:** Josef Finsterer, Claudia Stöllberger

**Affiliations:** ^1^Krankenanstalt Rudolfstiftung, 1030 Vienna, Austria; ^2^2nd Medical Department with Cardiology and Intensive Care Medicine, Krankenanstalt Rudolfstiftung, 1030 Vienna, Austria

## Abstract

*Objectives*. Presence of a factor-V Leiden mutation in a patient with myotonic dystrophy type 1 (DM1) has been reported only once. Here we report the second DM1 patient carrying a factor-V mutation who died from long-term complications of this mutation. *Case Report*. A 66-year-old DM1 patient with multi-organ-disorder syndrome developed a first deep venous thrombosis (DVT) and consecutive pulmonary embolism (PE) at age 50 y. Acetyl-salicylic acid was given. One year later he experienced a second DVT; that is why phenprocoumon was started. Despite anticoagulation, he experienced a third DVT bilaterally and a second PE bilaterally at 61 y; that is why a vena cava filter was additionally deployed. Despite therapeutic anticoagulation, he experienced a vena cava filter thrombosis at age 62 y. Genetic workup revealed a heterozygous factor-V mutation in addition to a CTG-repeat expansion of 500. As a consequence of PE he developed chronic obstructive pulmonary disease and experienced recurrent pulmonary infections, which were lastly responsible for decease at age 66 y despite intensive care measures. *Conclusion*. The heterozygous Leiden mutation may severely affect DM1 patients to such a degree that they die from its complications. If DM1 patients present with unusual manifestations, search for causes other than a CTG-repeat expansion is indicated.

## 1. Introduction

Double genetic troubles are rare or underdiagnosed and have been only rarely reported in patients with myotonic dystrophy type 1 (dystrophia myotonica (DM1)) [1, 2]. Here we present a DM1 patient with heterozygosity for the factor-V (Leiden) mutation, which lastly was responsible for the fatal outcome of this patient.

## 2. Case Report

**Figure 1:**
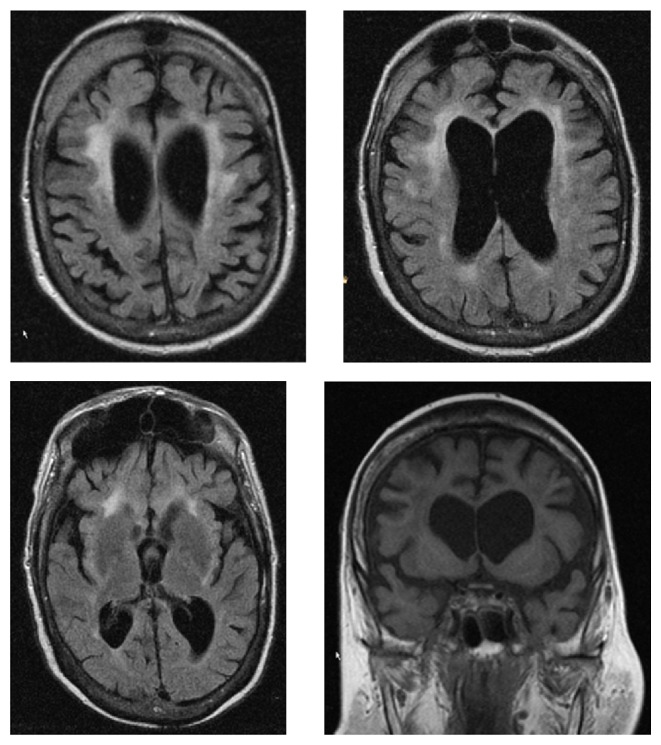
MRI of the cerebrum showing diffuse atrophy gliotic spots and leucoaraiosis. Pneumosinus dilatans can be also seen.

The patient is a 66- year-old Caucasian male, with height 176 cm and weight 77 kg, with a history of cholecystectomy at age 24 y, hypogonadotropic hypogonadism since age 26 y (onset of disease), and steatosis hepatis, hyperuricemia, and hyperlipidemia since at least age 42 y (Table 1). Since age 42 y, mild cognitive impairment developed. Since age 45 y, bilateral ptosis became apparent. Since at least age 49 y, hepatopathy was evident and he developed chronic obstructive pulmonary disease (COPD). At age 50 y, he experienced a first spontaneous deep venous thrombosis (DVT) with consecutive pulmonary embolism (PE). Acetyl-salicylic acid was given for secondary prophylaxis because of cognitive impairment. Cerebral CT (CCT) showed leukoencephalopathy and pneumosinus dilatans. At age 51 y, he experienced a second spontaneous DVT. Since then oral anticoagulation with phenprocoumon was given. Neurologic exam revealed ptosis exclusively. Diagnostic workup for the cause of recurrent DVT revealed the factor-V Leiden mutation in its heterozygous form (Table 2). Since age 53 y, weakness of the limb muscles developed. Muscle biopsy from the left brachial biceps muscle at age 54 y revealed only nonspecific alterations with some central nuclei and type-II-fiber predominance. Genetic testing at age 55 y revealed a heterozygous CTG-repeat expansion of 500 repeats in the* DMPK* gene on chromosome 19q13.3. Prostate hyperplasia became apparent and a synthetic growth factor was applied for growth retardation and short stature during 1 y, without success. At age 59 y, he experienced pneumonia two times. Thrombocytopenia became apparent (Table 1). Further downstream writing became increasingly impaired. Echocardiography showed aortic insufficiency grade 1 and mitral insufficiency grade 1. Cerebral MRI revealed marked general atrophy and marked leukoencephalopathy (Figure 1). At age 60 y, he experienced vestibular neuronitis but search for acoustic nerve neurinoma was negative. Clinical neurologic exam at age 60 y revealed myopathic face, bilateral hypoacusis, dysarthria, weak head anteflexion (M5-), diffuse weakness (M5-) of the upper limbs, cogwheel rigidity bilaterally, bilateral postural tremor, weakness for hip flexion (M5-), weakness for foot extension (M5-), general diffuse wasting, generally reduced tendon reflexes, and recurrent hyper-CKemia (Table 1). During hospitalisation, ascites from hepatopathy developed. At age 61 y, he experienced a second PE bilaterally with right-sided infarction pneumonia and a third DVT bilaterally. The INR value was outside the therapeutic range at this event. As a consequence, a vena cava filter was implanted. CCT showed atrophy and leukoencephalopathy. Abdominal CT revealed an atrophic pancreas, a left-sided renal cyst, and prostate hyperplasia. At age 62 y, he experienced thrombosis of the vena cava filter under an INR of 3.0. At age 63 y, he underwent cataract surgery bilaterally. At age 64 y, an AV-block I was documented for the first time. At age 65 y, an event recorder was implanted because of suspected arrhythmias. Recurrent interrogations revealed paroxysmal AFIB. The MMSE was 25. Shortly afterwards he was admitted to the intensive care unit because of severe dyspnea caused by pneumonia with an oxygen saturation of 63% and a pH of 6.92 requiring mechanical ventilation. Since hd1 he developed a striking restless-leg syndrome. On hd12 he underwent tracheostomy. On hd47 he received a percutaneous endoscopic gastrostomy (PEG). One day later he was transferred to the neurologic rehabilitation unit. Three days later he again required mechanical ventilation because of respiratory failure. He died on hd78 because of sepsis from pneumonia. The patient was a nonsmoker and nonalcoholic. His family history was positive for cataract and glaucoma (father), diabetes (brother of mother), and myocardial infarction (mother). He had one healthy son with gamma-globulin deficiency.

## 3. Discussion

The presented patient is interesting for several reasons. First, he is only the second patient with a double genetic trouble comprising a CTG-repeat expansion in the* DMPK* gene and the Leiden mutation [1]. The first DM1 patient with a Leiden mutation additionally had protein C deficiency [1]. In a further DM1 patient the CTG-repeat expansion was associated with a de novo shortened D4Z4 repeat fragment at the 4q35 locus giving rise to a facioscapulohumeral muscular dystrophy phenotype [3].

The second point of interest is that he experienced recurrent DVT despite only heterozygous APC resistance. He developed DVT and PE, despite oral anticoagulation and thus required a vena cava filter, and he developed thrombosis of the vena cava filter, despite sufficient anticoagulation. Possibly, the presence of the* DMPK* mutation enhanced the pathologic effect of the Leiden mutation. It can be speculated that expression of proteins required for the autochthon thrombolysis system was impaired to such a degree that it resulted in a gross prothrombotic effect. It is also possible that the mutated factor-V mRNA was further modified at the posttranslational level by sequestered RNA-binding proteins. It is also conceivable that liver disease favoured the development of DVT, PE, and cava filter thrombosis [4]. However, history and laboratory investigations were negative for hepatitis, HIV, cholangitis, cholestasis, and storage disease, and abdominal CT did not show liver disease. Secret alcohol consumption was rather unlikely given the absence of red blood cell stigmata. Thus, hepatopathy was most likely attributable to liver involvement in DM1. Though impaired mobility from DM1 has been reported to favour the development of thrombotic events [2] and the patient was lazy, it is rather unlikely that muscle involvement contributed to the etiology of recurrent DVT, since he displayed only mild muscle impairment, also in the leg compartment, and had rather normal mobility.

Third, the patient is interesting for marked central nervous system (CNS) involvement in the disease. He presented not only with dysarthria but also with cognitive decline, extrapyramidal manifestations, cogwheel rigidity, tremor, cerebral atrophy, and leukoencephalopathy, a combination of abnormalities previously described [5–8]. Of particular interest are the Parkinson's features (cogwheel rigidity, impaired writing) in the presented patient since, contrary to DM2 [9], only few DM1 patients have been reported with manifestations of Parkinson's syndrome [10].

Fourth, the patient is interesting for multiorgan disorder syndrome (MODS) affecting the skeletal muscles (ptosis, limb weakness, and weakness of respiratory muscles), the CNS (leukoencephalopathy, cognitive decline, dysarthria, cogwheel rigidity, postural tremor, impaired writing, and cerebral atrophy), the ears (hypoacusis, vestibular neuronitis), the liver (hepatopathy, steatosis, and cholecystolithiasis), the endocrine system (hypogonadism, prostate hyperplasia), the bones (pneumosinus dilatans [11]), and the immune system (reduced IgG, reduced IgM, and recurrent pulmonary infections) [12].

Fifth, the patient is the only DM1 patient reported receiving a vena cava filter. Whether this intervention favoured the development of recurrent pulmonary infections remains speculative. A further causative factor of pulmonary infections could be IgG deficiency, frequently found in DM1 patients, or IgM deficiency but also the recurrent PE resulting in COPD could be contributory. Additionally, it is possible that small PE occurred even after anticoagulation and placement of the vena cava filter, favouring recurrent pulmonary infections (infarction pneumonia). It is also conceivable, that PE had been mistaken as pneumonia. Since the patient never complained about dysphagia, microaspiration was rather excluded as the cause of recurrent pulmonary infections.

In conclusion, this case shows that a Leiden mutation, even if present in a heterozygous form, may severely affect DM1 patients to such a degree that they die from its long-term complications. If DM1 patients present with uncommon phenotypic manifestations, search for causes other than a CTG-repeat expansion including other mutations is indicated. Neurologists should be alert to the possibility that individuals having two separate mutations in unrelated genes may develop unusually severe phenotypes.

## Figures and Tables

**Table 1:** Blood chemical values.

Parameter	RL	6/99	7/01	7/02	2/07	10/07	11/07	2/08	12/09	12/11	4/12

Thrombocytes	150–300 G/L	nd	nd	nd	**90**	**99**	**108**	**111**	**124**	**117**	157
Uric acid	3.5–7.0 mg/dL	nd	**7.7**	6.6	nd	6.6	**7.3**	nd	nd	6.5	6.5
GOT	0–18 U/L	11	11	**28**	75	**59**	36	nd	39	**50**	36
GPT	0–23 U/L	11	11	14	**84**	**65**	**53**	nd	34	**79**	43
GGT	6–28 U/L	**47**	**32**	**32**	**217**	**181**	**158**	nd	**170**	**125**	**69**
TG	50–172 mg/dL	**299**	168	**180**	nd	**256**	**189**	**249**	**205**	**340**	**277**
CHE	150–200 mg/dL	**239**	**225**	**220**	nd	**200**	195	**255**	**225**	**256**	**320**
CK	0–80 U/L	39	60	nd	**351**	151	132	nd	**269**	**123**	136
IgG	700–1600 mg/dL	nd	nd	nd	nd	nd	**647**	nd	nd	nd	nd

RL: reference limits, GOT: glutamate-oxalate transaminase, GPT: glutamate-pyruvate transaminase, GGT: gamma-glutamyl transpeptidase, TG: triglycerides, CHE: cholesterol, CK: creatine-kinase, and nd: not done; since 2/07 other reference limits were used for GOT, GPT, GGT, and CK, and abnormal values are in bold.

**Table 2:** Coagulation parameters.

Parameter	RL	Result
Cardiolipin antibodies (IgG)	<18 GPL-U/mL	4.0
Cardiolipin antibodies (IgM)	<10 MPL-U/mL	0.8
Factor VIII:C	np	195
Factor IX	np	39
Factor XI	np	169
Factor XII	np	55
Protein C activity	np	63
Protein C immunological	np	70
Factor II immunological	np	64
Free protein S	np	43
APC resistance (ratio)	np	1.42
Factor-V Leiden	Negative	Heterozygote
Factor II mutation (n.20210G>A)	Negative	Negative
Homocysteine	np	20.6

RL: reference limits, np: not provided.
